# Predicting COVID-19 mortality risk in Toronto, Canada: a comparison of tree-based and regression-based machine learning methods

**DOI:** 10.1186/s12874-021-01441-4

**Published:** 2021-11-27

**Authors:** Cindy Feng, George Kephart, Elizabeth Juarez-Colunga

**Affiliations:** 1 Department of Community Health and Epidemiology, Faculty of Medicine, Dalhousie University, 5790 University Avenue, Halifax, B3H 1V7 NS Canada; 2 Department of Biostatistics and Informatics, University of Colorado Anschutz Medical Campus, 80045 Aurora, Colorado, 80045 USA

**Keywords:** COVID-19 mortality, Predictive model, Generalized additive model, Classification trees, Extreme gradient boosting

## Abstract

**Background:**

Coronavirus disease (COVID-19) presents an unprecedented threat to global health worldwide. Accurately predicting the mortality risk among the infected individuals is crucial for prioritizing medical care and mitigating the healthcare system’s burden. The present study aimed to assess the predictive accuracy of machine learning methods to predict the COVID-19 mortality risk.

**Methods:**

We compared the performance of classification tree, random forest (RF), extreme gradient boosting (XGBoost), logistic regression, generalized additive model (GAM) and linear discriminant analysis (LDA) to predict the mortality risk among 49,216 COVID-19 positive cases in Toronto, Canada, reported from March 1 to December 10, 2020. We used repeated split-sample validation and *k*-steps-ahead forecasting validation. Predictive models were estimated using training samples, and predictive accuracy of the methods for the testing samples was assessed using the area under the receiver operating characteristic curve, Brier’s score, calibration intercept and calibration slope.

**Results:**

We found XGBoost is highly discriminative, with an AUC of 0.9669 and has superior performance over conventional tree-based methods, i.e., classification tree or RF methods for predicting COVID-19 mortality risk. Regression-based methods (logistic, GAM and LASSO) had comparable performance to the XGBoost with slightly lower AUCs and higher Brier’s scores.

**Conclusions:**

XGBoost offers superior performance over conventional tree-based methods and minor improvement over regression-based methods for predicting COVID-19 mortality risk in the study population.

## Background

Coronavirus disease (COVID-19), caused by the severe acute respiratory syndrome coronavirus 2 (SARS-CoV-2), presents an unprecedented threat to global health worldwide. Cases have put a great burden on medical resources, leading to a shortage of intensive care resources. Prediction of mortality risk at the individual level is crucial for helping healthcare professionals prioritize medical care for patients by facilitating resource planning, and for guiding public health policy-making to mitigate the burden on the healthcare system.

For predicting event probability, logistic regression is commonly used. In logistic regression, linear effects are often assumed for continuous covariates, which may be restrictive in many applications. In contrast, generalized additive model (GAM) can model non-linear covariate effects [1–3]. For regression-based approaches, correct model specification is needed to ensure consistent probability estimates, which is challenging in the case of collinearity or complex interactive effects between independent variables.

To overcome these challenges, tree-based machine learning methods, such as classification tree [4], random forest [5], and gradient boosting [6, 7] have gained popularity in the literature. One advantage of tree-based methods is that they do not require specifying the parametric nature of the relationship between continuous predictors and the outcome. The tree-based methods can also easily handle categorical predictors without the need to create dummy variables. Further, tree-based methods allow for identifying high-risk sub-populations, especially when predictors have complex interaction effects. Nevertheless, the tree-based methods are prone to over-fitting, resulting in low bias but high variance, and limits to generalizability and robustness of models.

Research has been conducted comparing the performance of regression-based and tree-based methods in terms of predictive accuracy, but the results are inconclusive. Some studies concluded that classification tree and logistic regression had comparable performance [8, 9]; some studies concluded that logistic regression had superior performance over the tree-based methods [10, 11]; while some showed that tree-based methods outperform logistic regression [12–15]. One reason for this inconsistency is that comparative performance likely differs depending on the application and dimensionality of the data. Machine learning methods may perform better than regression-based methods when there are complex, contingent relationships between predictors, and data has high dimensionality. Thus, it is important to assess model performance for specific applications and data sources. Few studies have been conducted examining the use of machine learning methods for predicting COVID-19 mortality risk in Canada using available data sources.

If predictive models are going to be used for pandemic planning, validation to assess model robustness and performance is critical. In research on the performance of regression and machine learning methods, there is inconsistency in validation methods and how performance is assessed. Most studies used *k*-fold cross-validation (CV). Only a few employed repeated split-sample validation with a larger number of folds for CV to examine the robustness of the findings [8, 10]. Performance of the models at different levels of predicted probabilities is also important, as good performance overall may obscure predictive errors affecting those at different levels of risk.

The objective of this study was therefore to compare the performance of regression models and tree-based methods for predicting COVID-19 mortality in Toronto, Canada using data available in many settings. A range of individual and neighborhood-level predictors were considered. The predictive accuracy was assessed with repeated split-sample validation and forecast validation using the area under the receiver operating characteristic (ROC) curve and the Brier’s score. Predictive accuracy was also assessed at different levels of predicted probabilities.

## Methods

### Data description

Data on COVID-19 confirmed cases from March 1, 2020, through December 10, 2020, in the city of Toronto, Canada, were retrieved from the Ontario Ministry of Health. The outcome variable was an individual’s mortality status due to COVID-19. A range of predictors were considered. The COVID-19 epidemic is dynamic, increasing or decreasing over time, sometimes on a daily basis. It is therefore expected that time is an important predictor for COVID-19 mortality. In the dataset, the episode date, a derived/combined variable, was provided as the best estimate of when the disease was acquired and refers to the earliest available date from symptom onset (the first day that COVID-19 symptoms occurred), laboratory specimen collection date, or reported date. The time variable included in the predictive model is the elapsed days between the start date of the study (March 1, 2020) and the episode date. The demographic characteristics of the subject include age groups: ≤19, 20-29, 30-39, 40-49, 50-59, 60-69, 70-79, 80-89, 90+ years of old and self-reported gender: males, females and others, where others represent unknown or other sexual identifications such as transgender. Toronto is divided into 140 geographically distinct neighborhoods that were established to help government and community agencies with local planning by providing meaningful social and economic ecological data from census and other sources. Neighborhood-level predictors over the 140 neighborhoods in Toronto were obtained from the 2016 Canadian Census data, including population density and average household income, which were linked to the COVID-19 data by neighborhoods. Research shows that temperature is negatively associated with COVID-19 transmission [16, 17]. Therefore, the daily temperature in Toronto from March 1, 2020, until December 10, 2020, was included as a predictor, downloaded from the Government of Canada Daily Weather Data Report. Variables describing the history of hospitalization for COVID-19 (ever hospitalized, ever in the intensive care unit (ICU), or ever intubated) were also used as predictors. These variables may be intermediate outcomes between infection and death, and may interact with other variables as predictors of mortality. For example, individual and neighborhood variables may be proxies for health status and chronic disease variables associated with serious COVID-19 outcomes. They may also be associated with differences in health care access and quality, and thus modify the relationship between intermediate hospital outcomes and death.

### Predictive models for COVID-19 mortality risk

#### Regression methods

##### Logistic Regression

The logistic regression (LR) model with the logit link function can be expressed as, logit(*π*_*i*_)=***X***_*i*_***β***, where *π*_*i*_ denotes the probability of mortality and ***X***_*i*_ is the design matrix for all the covariates and ***β***=(1,*β*_1_,...,*β*_*p*_)^*T*^ is a *p*×1 vector of regression coefficients. We considered two types of logistic regression models. The first model consisted of all the variables. No variable reduction was performed. In the second model, all the variables and their two-way interactions were included in the initial model. Then, the Least Absolute Shrinkage and Selection Operator (LASSO) [18] was used to exclude “unnecessary” predictors by shrinking their coefficients to exactly zero, yielding a more parsimonious model. The hyperparameter or regularization parameter controlling the amount of regularization in the LASSO regression is chosen by minimizing misclassification error in terms of Area Under the ROC curve (AUC) based on 10 fold cross-validation. The function cv.glmnet in the glmnet package [19] in R was used for implementing the LASSO method.

##### Generalized Additive Models

Generalized additive models (GAMs) are a non-parametric, regression technique providing greater flexibility in modeling non-linear covariate effects with smoothed splines [1, 20], which can be described as $\text {logit}(\pi _{i})=\boldsymbol {X}_{i}\boldsymbol {\beta }+\sum _{j=1}^{J}f_{j}(z_{ij})$ where ***X***_*i*_ is a row of the design matrix for any parametric model component, such as age groups, gender and critical care use; ***β*** is the corresponding parameter vector; *f*_*j*_(*z*_*ij*_) denote non-parametric spline functions for the *j*th continous predictor, *j*=1,⋯,*J*, respectively. A penalized log-likelihood method is maximized to estimate all the parameters [1]. The smoothing parameters are estimated by the generalized cross-validation method [20]. The above model is fitted using the R package mgcv [20].

##### Linear Discriminant Analysis

Linear discriminant analysis (LDA) [21] models the distribution of the predictors separately in each of the response classes and then uses Bayes’ theorem to convert these back into estimates for the probability of an event. When the response variable classes are well-separated, logistic regression may be unstable, but LDA does not suffer from this problem. However, LDA assumes the distribution of the predictors *X* are approximately normal in each of the classes and have a common variance, which may fail to hold in some cases. DA has closed-form solutions, so it has no hyperparameters to tune. LDA was implemented in the lda function in the R package MASS [22].

#### Tree-based methods

##### Classification Tree

Classification tree has become a popular alternative to logistic regression [4]. Unlike logistic and linear regression, a classification tree does not develop a prediction equation. The method firstly partitions the sample into two distinct samples according to all possible dichotomizations of all continuous variables given a threshold, and all the categorical variables. Then, the partition that yields the greatest reduction in impurity is selected. The procedure is then repeated iteratively until a pre-specified stopping rule is met. After the entire feature space is split into a certain number of simple regions recursively, the predicted probability of the event for a given subset can be calculated using the proportion of subjects who have the condition of interest among all the subjects in the subset to which the given subject belongs [23].

In this study, the classification tree model was implemented using the R package rpart [24]. At each node, the partition was chosen that maximized the reduction in misclassification error. The minimum number of observations that must exist in a node in order for a split to be attempted was 30. The maximum depth of any node of the final tree was 100. The value of the complexity parameter (cp) was set as cp =0.001. Any split that did not decrease the overall lack of fit by a factor of cp was not attempted. To reduce the variance of the resulting models and prevent overfitting the data, the trees were then pruned by removing any split which did not improve the fit. The optimal size of each tree was determined using cross-validation using the cptable function, which selects the optimal cp with lowest cross validation error. Pruning the tree was done using the prune function of the rpart R package.

##### Random Forest

Classification trees tend to overfit the training dataset, which may lead to low bias, but high variance [25]. To remedy the issue of high variation in classification trees, the results from multiple trees based on bootstrap samples from the original data can be aggregated, which are referred to as ensemble methods. A common ensemble method with trees is the random forest (RF) approach [26], which is a bagging procedure to combine multiple trees based on bootstrap samples from the original data. One tree is built from each bootstrap sample by introducing recursive binary splits to the data. At a given node, rather than considering all possible binary splits on all candidate predictors, it only considers a random sample of the candidate predictor variables to lower the correlation between trees.

For this study, 1000 regression trees were grown, and the size of the set of randomly selected predictor variables used for determining each binary split was the square root of the number of predictor variables (rounded down), which is the default parameter value in the R package randomForest [5]. In contrast to the classification tree, trees of an RF are not pruned back.

##### Extreme Gradient Boosting

Gradient boosting tree is an ensemble method of classification trees by iteratively refitting weak classifier to residuals of previous models, meaning that the current weak classifier was generated based on the previous one to optimize the predictive efficiency [6, 7]. Extreme gradient boosting (XGBoost) is an efficient implementation of the gradient boosting method [27], which can learn nonlinear relations among input variables and outcomes in a boosting ensemble manner to capture and learn nonlinear and complex relations accurately. Extreme gradient boosting can improve the accuracy of a classification tree [12–15].

In this study, XGBoost was implemented using the xgboost package in R, which automatically does parallel computation on a single machine, and is thus more computationally efficient than other gradient boosting packages. Hyperparameter optimization was performed to prevent overfitting of the model on the training data. Due to computational and time constraints, hyperparameter optimization was performed across a sparse parameter grid to determine the optimal combination of candidate hyperparameters, i.e., depth of the tree 1,2,3,4,5,6, shrinkage factor =0.01,0.02,0.03,0.04,0.05, and the maximum number of iterations= 500,1000,1500,2000.

### Predictive model assessment

#### Cross validation

##### Repeated Split-Sample Validation

Repeated split-sample validation [10] was used to compare the predictive accuracy of each statistical method. The data were randomly divided into 80% training and 20% testing datasets. Each model was fit on the training dataset. Predictions were then obtained in the testing dataset using the model derived from the training dataset. This process was repeated 200 times, i.e., each predictive model was fit using the training dataset. The model was then used to predict the mortality risk based on the testing dataset. Results were then summarized over the 200 testing datasets. Repeated split-sample validation assesses the robustness of the results and is less likely to be impacted by influential observations in only a few testing samples.

##### Forecasting Validation

We also validated the models based on the *k*-step-ahead predictions of the last *k* days of the observation period, where *k*=7,8,⋯,30. For each of the *k*-step-ahead predictions, the training dataset was all the data prior to the *k* days to be predicted. Each model fit the training dataset, and predictions were obtained for the last *k* days of the testing dataset.

#### Performance measures

Discrimination of the prediction method can be measured by the area under the ROC curve (AUC) [28]. Higher values of the AUC indicate better model discrimination. AUC examines the ability of the method to distinguish whether the patients who have the outcome have higher risk predictions than those who do not, but does not account for calibration, i.e., the magnitude of the disagreement between the observed and predicted responses [28]. To quantify how close the predictions are to the actual outcome, Brier’s score [28, 29] was used, which is defined as, $1/n \sum _{i=1}^{n} (\hat {\pi }_{i}-Y_{i})^{2}$, where $\hat {\pi }_{i}$ is the predicted probability in the testing set, and *Y*_*i*_ is the observed response for the *i*th subject in the testing set. Lower Brier’s scores indicate greater model accuracy. Performance was further quantified using calibration measurement, which fits a logistic regression to model the outcome variable against the logit of the predicted probabilities as the independent variable in the testing dataset. For a well calibrated prediction model, the intercept of the calibration model should be zero and the slope should be one. We also assessed the models by graphically comparing the agreement of the predicted versus observed probabilities over the range of the predicted probabilities.

## Results

### Description of the study sample

The study sample includes *n*=49,216 COVID-19 positive cases, of whom 1938 (3.9%) died from COVID-19. Comparison of the sample characteristics by patients’ mortality status due to COVID-19 is reported in Table 1. The neighborhood-level variables (population density and average household income) and the daily mean temperatures are continuous predictors, which may have a nonlinear relationship with the COVID-19 mortality risk. In Table 1, these variables were categorized into four categories to describe their distributions in relation to COVID-19 mortality status; however, in the predictive models, all these variables are modeled as continuous predictors. The results presented in Table 1 show statistically significant differences in all the predictors between COVID-19-infected individuals who died versus not.

**Table 1:** Characteristics of the study sample by COVID-19 mortality status

	All	Alive	Died	*P*-value
	*n*=49216	*n*=47278	*n*=1938	
Age				<0.001
19 and younger	5439 (11.1%)	5438 (100.0%)	1 (0.02%)	
20 to 29 Years	9635 (19.6%)	9634 (100.0%)	1 (0.01%)	
30 to 39 Years	7889 (16.0%)	7887 (100.0%)	2 (0.03%)	
40 to 49 Years	6955 (14.1%)	6944 (99.8%)	11 (0.16%)	
50 to 59 Years	7161 (14.6%)	7101 (99.2%)	60 (0.84%)	
60 to 69 Years	4684 (9.52%)	4511 (96.3%)	173 (3.69%)	
70 to 79 Years	2593 (5.27%)	2257 (87.0%)	336 (13.0%)	
80 to 89 Years	2854 (5.80%)	2166 (75.9%)	688 (24.1%)	
90 and older	2006 (4.08%)	1340 (66.8%)	666 (33.2%)	
Gender				0.002
Female	25192 (51.2%)	24205 (96.1%)	987 (3.92%)	
Male	23670 (48.1%)	22746 (96.1%)	924 (3.90%)	
Unknown	354 (0.72%)	327 (92.4%)	27 (7.63%)	
Ever Hospitalized				<0.001
No	45604 (92.7%)	44663 (97.9%)	941 (2.06%)	
Yes	3612 (7.34%)	2615 (72.4%)	997 (27.6%)	
Ever in ICU				<0.001
No	48500 (98.5%)	46853 (96.6%)	1647 (3.40%)	
Yes	716 (1.45%)	425 (59.4%)	291 (40.6%)	
Ever Intubated				<0.001
No	48770 (99.1%)	47044 (96.5%)	1726 (3.54%)	
Yes	446 (0.91%)	234 (52.5%)	212 (47.5%)	
Population Density:				<0.001
(1.04e+03,3.2e+03)	12461 (25.3%)	11893 (95.4%)	568 (4.56%)	
(3.2e+03,4.89e+03)	12274 (24.9%)	11715 (95.4%)	559 (4.55%)	
(4.89e+03,7.2e+03)	12889 (26.2%)	12470 (96.7%)	419 (3.25%)	
(7.2e+03, 44.32e+03)	11592 (23.6%)	11200 (96.6%)	392 (3.38%)	
Average Income:				<0.001
(2.38e+04,2.75e+04)	12490 (25.4%)	12196 (97.6%)	294 (2.35%)	
(2.75e+04,2.98e+04)	12964 (26.3%)	12446 (96.0%)	518 (4.00%)	
(2.98e+04,3.79e+04)	11503 (23.4%)	10967 (95.3%)	536 (4.66%)	
(3.79e+04, 19.34e+04)	12259 (24.9%)	11669 (95.2%)	590 (4.81%)	
Daily Mean Temperature				<0.001
(-8.9,-1.2)	1937 (3.94%)	1879 (97.0%)	58 (2.99%)	
(-1.2,0.7)	3081 (6.26%)	2980 (96.7%)	101 (3.28%)	
(0.7,8.4)	19772 (40.2%)	18672 (94.4%)	1100 (5.56%)	
(8.4, 28.4)	24426 (49.6%)	23747 (97.2%)	679 (2.78%)	

We included two different sets of predictors in the models. The first set included all individual and neighborhood variables, and also variables describing hospital use for COVID-19 conditions (ever hospitalized, ever in ICU and ever intubated). Hospitalization, ICU use and intubationtare often intermediate outcomes between infection and mortality, and may interact with individual and neighborhood variables in predicting mortality as a result of differences in risk (e.g. due to health status and chronic disease) and quality of care. The second set of predictors included only individual and neighborhood variables, thus omitting intermediate hospital outcomes as predictors of mortality.

### Comparison of predictive ability of predictive methods

#### Repeated Split-Sample validation

**Fig. 1:**
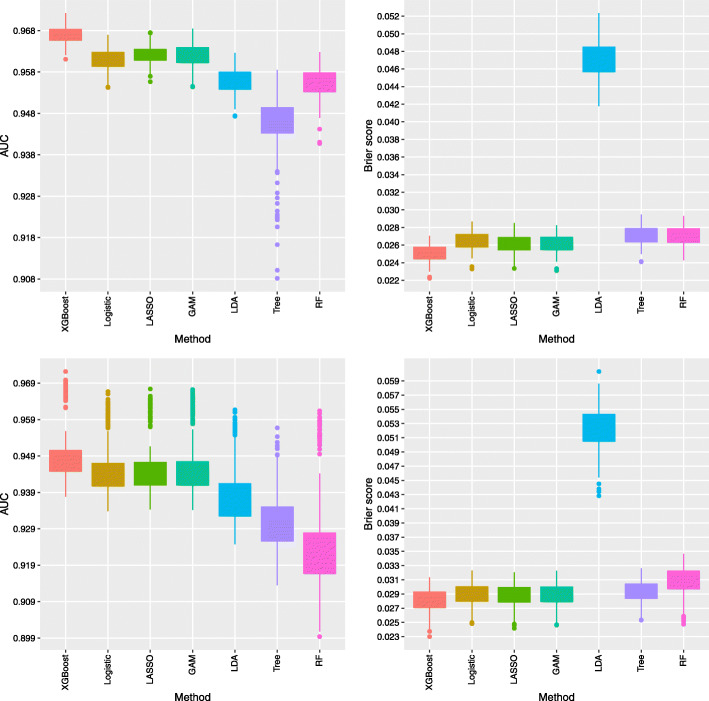
AUC (left panels) and Brier’s score (right panels) over the 200 testing samples of the repeated split sample validation for the methods including all the considered predictors (top panels) and excluding hospital use variables (ever hospitalized, ever ICU use and ever intubated) for COVID-19 as predictors (bottom panels)

The predictive accuracy of the methods averaged over 200 repeated split samples are reported in Table 2. The results indicate XGBoost yields the highest AUC at 0.9669 and the lowest Brier’s score at 0.0251. The regression-based methods (logistic, LASSO, and GAM) perform almost equivalently well as XGBoost at only slightly lower AUCs (0.9610 to 0.9622) and higher Brier’s scores (0.0261 to 0.0265). LDA results in a lower predictive accuracy with AUC at 0.9559 and the highest Brier’s score at 0.0471. Among the tree-based methods, the classification tree yields the lowest AUC at 0.9450 and the highest Brier’s score at 0.0271. RF provides an improvement over the classification tree with a higher AUC value at 0.9552 and a lower Brier’s score at 0.0270. However, both classification tree and RF methods do not perform as well as the XGBoost method. Excluding history of hospital use for COVID-19 conditions as predictors results in worse predictive accuracy for all type of models. Nevertheless, the relative performance of the methods is consistent with the results when including hospital use as predictors. For ease of comparison, the distributions of the AUC and Brier’s test scores over the 200 repeated samples for all the methods are displayed in Fig. 1.

**Table 2:** Comparison of model performance in terms of AUC, Brier’s score, calibration intercept and calibration slope averaged over the 200 testing samples in the repeated split-sample validation

Methods	Predictive Accuracy		Calibration	
	AUC	Brier	Intercept	Slope
	Including Hospital Use Variables			
logistic	0.9610	0.0265	-0.0303	0.9827
LASSO	0.9622	0.0261	-0.0109	0.9958
GAM	0.9620	0.0262	-0.0620	0.9592
LDA	0.9559	0.0471	-1.6859	0.3630
Tree	0.9450	0.0271	-0.0893	0.9378
RF	0.9552	0.0270	-0.2993	0.5798
XGBoost	0.9669	0.0251	0.0464	1.0287
	Excluding Hospital Use Variables			
logistic	0.9423	0.0296	-0.0179	0.9879
LASSO	0.9424	0.0295	0.0145	1.0087
GAM	0.9425	0.0295	-0.0474	0.9692
LDA	0.9348	0.0536	-1.6425	0.4178
Tree	0.9276	0.0299	-0.0463	0.9697
RF	0.9190	0.0317	-0.7027	0.4783
XGBoost	0.9461	0.0288	0.0235	1.0090

In the calibration assessment (Table 2), XGBoost and LASSO have a calibration intercept closest to zero and calibration slope closest to one as compared to the other methods. Logistic and GAM result in a slightly worse calibration compared to XGBoost and LASSO. Of the tree-based methods, RF has much worse calibration as compared to the classification tree, and both are not comparable with the XGBoost method. LDA has the worst performance in terms of calibration.

**Fig. 2:**
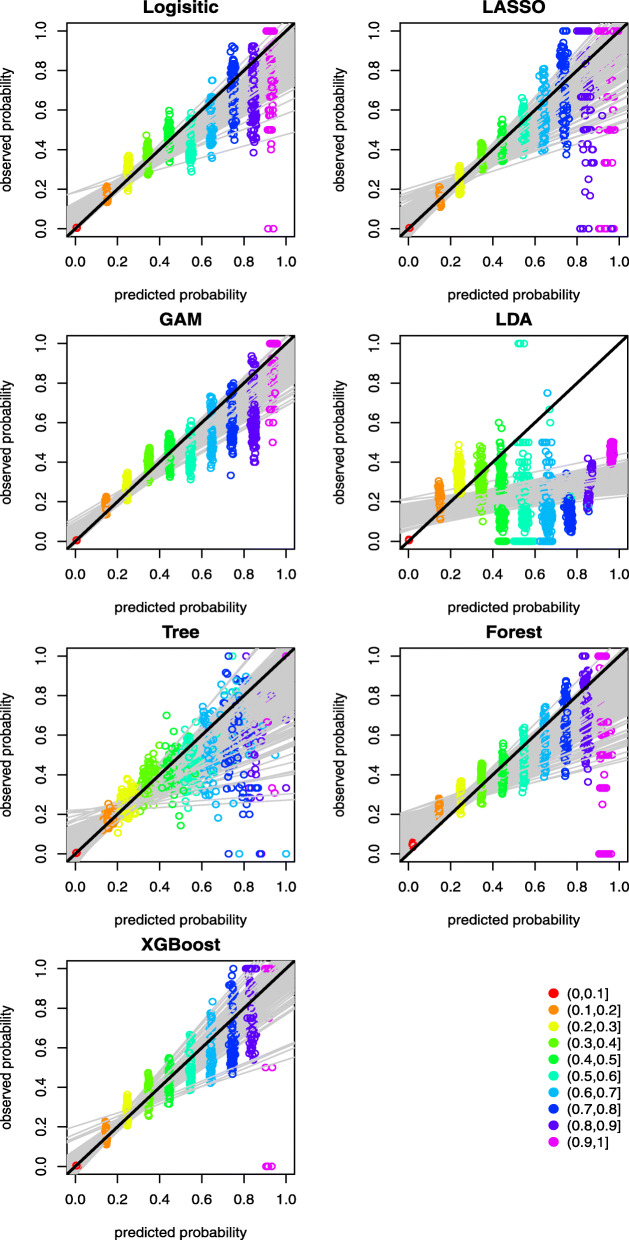
Mean predicted probability versus the observed event rate over 10 predicted probability groups between 0 and 1 at an increment of 0.1 for the scenario with hospital use for COVID-19 predictors. The black line at 45 degree represents a line of perfect calibration. The grey lines are the linear regression lines for modelling the observed event rate against the mean predicted probabilities over the 10 groups as predictor

**Fig. 3:**
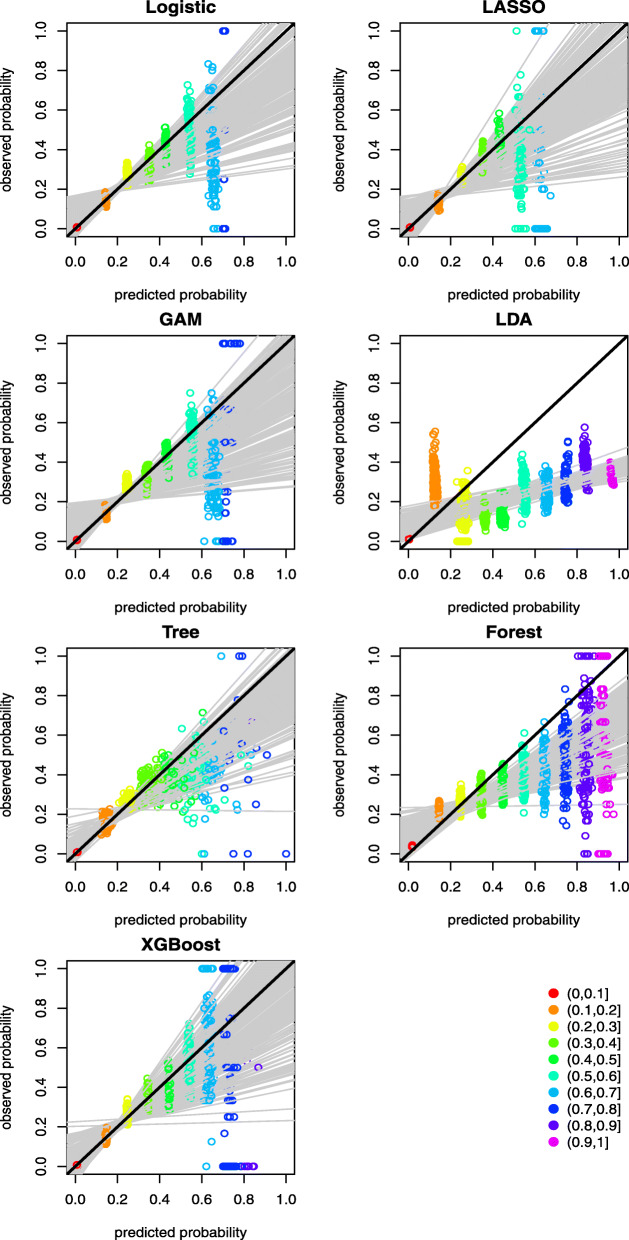
Mean predicted probability versus the observed event rate over 10 predicted probability groups between 0 and 1 at an increment of 0.1 for the scenario without hospital use for COVID-19 as predictors. The black line at 45 degree represents a line of perfect calibration. The grey lines are the linear regression lines for modelling the observed event rate against the mean predicted probabilities over the 10 groups as predictor

A graphical assessment of calibration presents predictions on the x-axis, and the outcome on the y-axis [30]. Perfect predictions are on the 45-degree line. Further, examining calibration at various levels of predictive probability provides additional insights of the agreement between predicted and observed mortality risk. Ideally, a calibration measure would compare the predicted probability with the true probability for each individual, but the measurement of actual probability for a single individual is challenging. Forming groups of individuals and calculating the proportion of positive outcomes is an approach to calculating the observed or true probability of an event or outcome, which is the central idea of the Hosmer-Lemeshow (H-L) test [31]. There are two popular ways of grouping individuals: (1) group using deciles of predicted probability, and (2) group using equal intervals according to the predicted probability. We adopted the latter grouping method to graphically demonstrate the calibration of the predictive methods at various levels of predictive probability. This is achieved by splitting the individuals into 10 equally spaced groups between 0 and 1 according to their predicted probabilities of COVID-19 mortality. Model calibration can then be assessed graphically by plotting the mean predicted versus observed event rates for the 10 groups, thus providing information on the direction or magnitude of miscalibration [30]. The results are presented in Figs. 2 and 3 for the case with and without history of hospital use for COVID-19, respectively. The graphs reveal that the points in the lower risk intervals are closer to the 45-degree diagonal line. By contrast, the points in the higher risk intervals are more dispersed, which can be explained by the fact that very few patients had predicted risk above 0.8 and the prediction above this threshold appears to be less well-calibrated. Most of the methods suffer from the over-prediction of risk in the high-risk groups. XGBoost appears to provide better calibration with points more closely distributed around the 45-degree diagonal line across the groups. When hospital use for COVID-19 variables are not included as predictors, the predicted probabilities are mostly below 0.8, as shown in Fig. 3. This indicates the distributions of predicted risk of mortality are less spread out compared to models that omit hospital use variables as predictors.

**Fig. 4:**
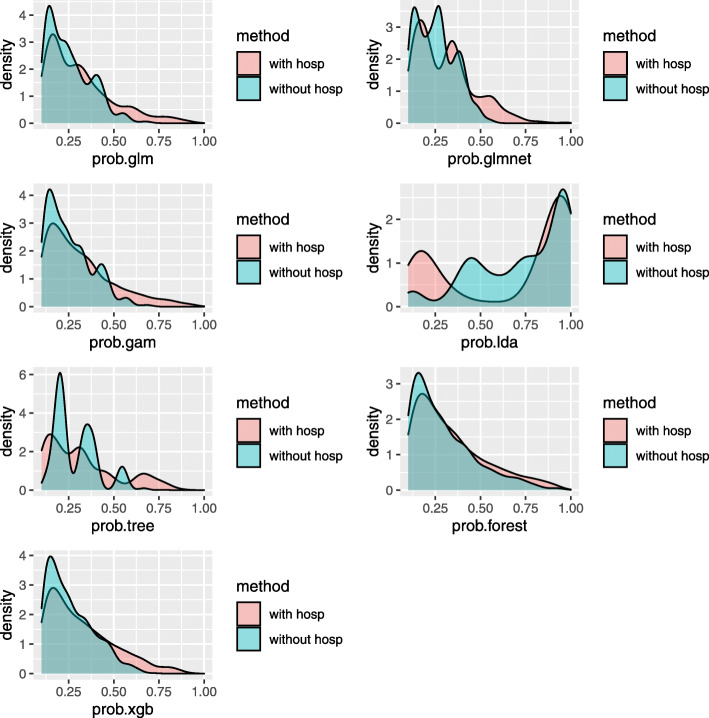
Density plots of the predicted mortality probability based on a random sample of the 200 repeated split samples. The distributions of the predictive mortality probability are highly right-skewed, so the predicted probabilities below 0.2 are suppressed for better visualization of the higher predictive risk

A better discriminating model has more dispersed predictive probabilities than a poorly discriminating model. Therefore, the distributions of the predicted mortality probability based on a random sample of the 200 repeated split samples for all the methods are displayed in Fig. 4. The distribution of the predicted mortality probability based on all the repeated split samples yielded very similar results, so only one random sample is presented for simplicity of illustration. The distributions of the predictive mortality probability are highly right-skewed, so the predicted probabilities below 0.2 are suppressed for better visualization of the higher predictive risk. As shown in Fig. 4, all of the predictive methods with hospital use variables as predictors, except for LDA method, had longer right tails in the predicted mortality probability compared to the counterpart models that omit hospital use variables as predictors. Therefore, it is expected that the methods including hospital use variables as predictors have better discrimination and calibration performance compared to the methods omitting hospital use variables as predictors.

**Fig. 5:**
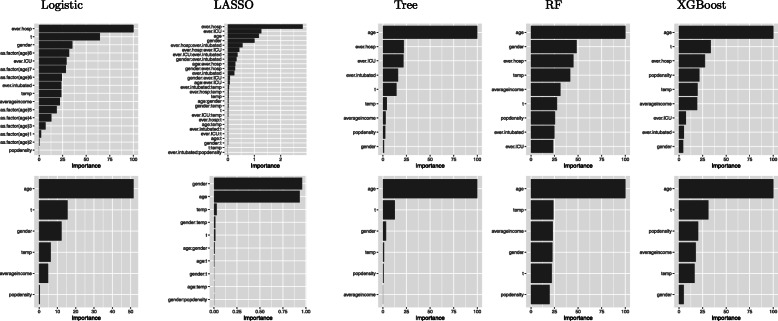
Variable importance in the prediction of COVID-19 mortality risk with (top panels) and without (bottom panels) hospital use for COVID-19 predictors using the R package vip. From left to right, the panels are for Logistic, LASSO, Tree, RF and XGBoost, respectively

Evaluating differences in the importance of predictors provides additional insight into model differences. The importance of predictors in order of significance with and without history of hospital use for COVID-19 variables are presented in Fig. 5. Predictors with the largest influence varied considerably between the different methods. For the XGBoost method, age is the strongest predictor, followed by reporting time. Of history of hospital use variables, ever in hospital is the strongest predictor. The neighborhood-level factors (population density and average income) and temperature, also contribute to the prediction. Gender has the least contribution to the prediction.

#### Forecasting validation

The predictive accuracy of all methods for predicting daily COVID-19 mortality risk over the last 7 to 30 days of the observational period is reported in Table 3. Notably, compared to repeated split-sample validation, the predictive accuracy of all the methods for forecasting, as measured by AUC, tends to be higher. XGBoost yields the highest AUC of 0.9866 and the lowest Brier’s score of 0.0091. The regression-based methods (logistic, LASSO and GAM) again perform nearly equivalently well as the XGBoost method with AUC ranging from 0.9819 to 0.9842 and Brier’s score ranging from 0.0094 to 0.0096, with LASSO being the method most comparable to XGBoost. Among the tree-based methods, the classification tree results in the lowest AUC value at 0.9781 and highest Brier’s score at 0.0098. RF improved over the classification tree with a higher AUC at 0.9808 and a lower Brier’s score at 0.0096. Despite the higher AUC values for forecasting CV compared to repeated-split sample CV, the calibration for forecasting CV tends to be poorer compared to repeated-split sample CV.

**Table 3:** Comparison of model performance in terms of AUC, Brier’s score, calibration intercept and slope averaged over the last 7 to 30 days at the end of the observational period in the forecasting validation

Methods	Predictive Accuracy		Calibration	
	AUC	Brier	Intercept	Slope
	Including hospital use variables			
logistic	0.9819	0.0094	0.1031	1.2468
LASSO	0.9842	0.0096	-0.2558	1.1376
GAM	0.9823	0.0095	0.4267	1.2265
LDA	0.9849	0.0172	-2.1364	0.5276
Tree	0.9781	0.0098	-0.1898	1.0332
RF	0.9808	0.0096	-0.2993	0.5798
XGBoost	0.9866	0.0091	0.4799	1.2934
	Excluding hospital use variables			
logistic	0.9472	0.0121	0.0712	1.0800
LASSO	0.9453	0.0121	-0.1516	0.9540
GAM	0.9473	0.0121	0.1983	1.0987
LDA	0.9331	0.0185	-1.5685	0.5427
Tree	0.9133	0.0124	-0.2133	1.0171
RF	0.9229	0.0130	-1.0875	0.6017
XGBoost	0.9487	0.0123	0.1079	1.0421

**Fig. 6:**
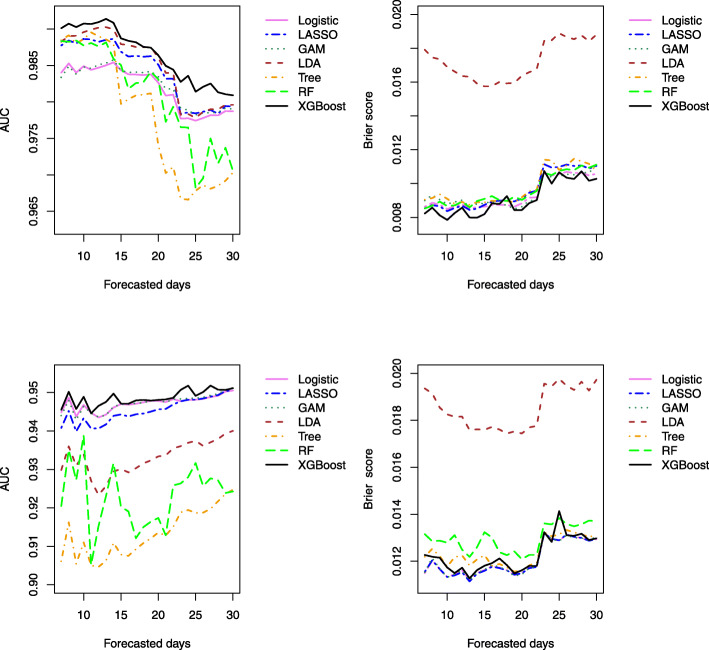
AUC and Brier’s score for forecasting validation based on the *k*-steps-ahead prediction of the last *k* days of the observation period *k*=7,8,⋯,30. The top and bottom panels are based on the methods including (top panels) and excluding (bottom panels) hospital use (ever hospitalized, ever ICU and ever intubated related to COVID-19) as predictors, respectively

The predictive ability of the methods for forecasting mortality risk for the last 7 to 30 days at the end of the observational period is displayed graphically in Fig. 6. The results indicate that the accuracy of all the methods tends to decrease as the number of forecast days increases. XGBoost consistently outperforms the other methods over the forecasting time window. Interestingly, the superior performance of XGBoost over the regression-based methods in terms of AUC is more substantial in the scenario when the history of hospital use predictors are included, compared to the scenario when they are omitted. This indicates the hospital use predictors may have complex interactive effects with the rest of the predictors for predicting the mortality risk. By contrast, in the scenario omitting hospital use predictors, logistic regression performs equivalently to XGBoost. In this case with only a few predictors being considered, the advantage of XGBoost to identify complex relationships between input variables and the outcomes is less pronounced.

## Discussion

This article compared regression and tree-based machine learning methods for predicting COVID-19 mortality risk in Toronto, Canada. This investigation demonstrates that predictive models based on machine learning methods, applied to available data, can provide important insights to inform resource planning for health care services to address the burden of the COVID-19 pandemic.

Our findings revealed that using machine learning methods to data employing a few easily accessible predictor variables, including age, hospital use for COVID-19, episode date, gender, and neighborhood demographic and economic characteristics, it is possible to predict the risk of COVID-19 mortality with a high degree of predictive power. Our findings also provide insight into the best choice of machine learning methods to use. We found that XGBoost outperforms the conventional regression tree methods, probably because it is a regularized model formalization to control over-fitting. We fit three separate logistic regression models: main effect only, GAM and LASSO. The LASSO’s predictive performance is slightly better than the main effect only method, which indicates interactions among some predictors may exist. Compared to the logistic regression, GAM yielded an almost identical model fit, which implies that assuming linear relationships between input variables and the outcomes might be adequate in this study. However, note that we did not include two-way interactions in the GAM method due to model fitting complexity. For this reason, concluding the appropriateness of the linearity assumption may be premature. In this study, we only considered a few predictors. As the number of correlated and interactive predictors increases, LASSO would likely outperform the other regression-based methods. When non-linear covariates effects are pronounced, GAM is expected to outperform the conventional logistic regression methods. LDA resulted in the worst predictive accuracy in this study, which indicates the assumptions of LDA do not hold (i.e., Predictors in this study are likely not drawn from a Gaussian distribution with a common covariance matrix in each class).

There are limitations to this study that merit discussion. One major limitation of this study is the unavailability of data on clinical characteristics of patients, such as co-morbidities. Recent research has identified certain chronic health conditions risk factors (e.g. obesity) as strong predictors of prognosis and severity of progression for COVID-19 [32]. These crucial pieces of information are not readily available in publicly accessible data, but could be obtained from administrative health databases. Another potential limitation is the inclusion hospitalization, ICU use, and intubation for COVID-19 as predictors. While they are clearly important predictors, the interpretation of these predictors and the policy implications of including them in models need to be considered. They may be proxies for patients’ underlying health status, or proxies for access to and quality of care. They are also intermediate health outcomes prior to most COVID-19 deaths. Another limitation is that we did not consider support vector machine techniques or neural networks [25], which could be alternative approaches for predicting COVID-19 mortality risk.

Despite the limitations, our findings revealed that by focusing on a few easily accessible variables, including age, past hospital use for COVID-19, episode date, gender, and neighborhood demographic and economic characteristics, it is possible to predict the risk of mortality with high predictive power in the studied population.

## Conclusion

The study demonstrates that the high predictive accuracy for COVID-19 mortality risk can be achieved based on publicly available data in the studied population. This study provided a careful assessment of the predictive accuracy of the regression and tree-based machine learning methods for predicting COVID-19 mortality risk among confirmed cases in the study region. Although the prediction model established in our study included only a few easily accessible variables, XGBoost and LR-based methods have high predictive power with XGBoost resulting in slightly better performance. This type of data-driven risk prediction may assist health resource planning for COVID-19.

## Data Availability

The datasets generated and/or analysed during the current study are available in the City of Toronto’s Open Data Portal, https://open.toronto.ca/

## Abbreviations

COVID-19: coronavirus disease
XGBoost: extreme gradient boosting
GAM: generalized additive model
LDA: linear discriminant analysis
LASSO: least absolute shrinkage and selection operator
Tree: classification tree
RF: random forest
AUC: area under the receiver operating characteristic (ROC) curve ICU: intensive care unit CV: cross validation
